# Metabolomic analysis of dietary‐restriction‐induced attenuation of sarcopenia in prematurely aging DNA repair‐deficient mice

**DOI:** 10.1002/jcsm.13433

**Published:** 2024-04-30

**Authors:** Yupeng He, Wei Yang, Luojiao Huang, Marlien Admiraal‐van Mever, Rawi Ramautar, Amy Harms, Yvonne Rijksen, Renata M.C. Brandt, Sander Barnhoorn, Kimberly Smit, Dick Jaarsma, Peter Lindenburg, Jan H. J. Hoeijmakers, Wilbert P. Vermeij, Thomas Hankemeier

**Affiliations:** ^1^ Metabolomics and Analytics Centre, Leiden Academic Centre for Drug Research Leiden University Leiden The Netherlands; ^2^ Princess Máxima Center for Pediatric Oncology Utrecht The Netherlands; ^3^ Oncode Institute Utrecht The Netherlands; ^4^ Department of Molecular Genetics, Erasmus MC Cancer Institute Erasmus University Medical Center Rotterdam Rotterdam The Netherlands; ^5^ Department of Neuroscience Erasmus University Medical Center Rotterdam Rotterdam The Netherlands; ^6^ Research Group Metabolomics, Leiden Center for Applied Bioscience University of Applied Sciences Leiden Leiden The Netherlands; ^7^ Institute for Genome Stability in Aging and Disease, Cologne Excellence Cluster for Cellular Stress Responses in Aging‐Associated Diseases (CECAD) University of Cologne Cologne Germany

**Keywords:** Dietary restriction, Energy generation, Inflammation, Muscle aging, Oxidative stress, Progeria

## Abstract

**Background:**

Sarcopenia is characterized by loss of skeletal muscle mass and function, and is a major risk factor for disability and independence in the elderly. Effective medication is not available. Dietary restriction (DR) has been found to attenuate aging and aging‐related diseases, including sarcopenia, but the mechanism of both DR and sarcopenia are incompletely understood.

**Methods:**

In this study, mice body weight, fore and all limb grip strength, and motor learning and coordination performance were first analysed to evaluate the DR effects on muscle functioning. Liquid chromatography–mass spectrometry (LC–MS) was utilized for the metabolomics study of the DR effects on sarcopenia in progeroid DNA repair‐deficient *Ercc1* ^*∆/*−^ and *Xpg* ^*−/−*^ mice, to identify potential biomarkers for attenuation of sarcopenia.

**Results:**

Muscle mass was significantly (*P* < 0.05) decreased (13–20%) by DR; however, the muscle quality was improved with retained fore limbs and all limbs grip strength in *Ercc1* ^*∆/*−^ and *Xpg* ^*−/−*^ mice. The LC–MS results revealed that metabolites and pathways related to oxidative‐stress, that is, GSSG/GSH (*P* < 0.01); inflammation, that is, 9‐HODE, 11‐HETE (*P* < 0.05), PGE_2_, PGD_2_, and TXB_2_ (*P* < 0.01); and muscle growth (PGF_2α_) (*P* < 0.01) and regeneration stimulation (PGE_2_) (*P* < 0.05) are significantly downregulated by DR. On the other hand, anti‐inflammatory indicator and several related metabolites, that is, β‐hydroxybutyrate (*P* < 0.01), 14,15‐DiHETE (*P* < 0.0001), 8,9‐EET, 12,13‐DiHODE, and PGF_1_ (*P* < 0.05); consumption of sources of energy (i.e., muscle and liver glycogen); and energy production pathways, that is, glycolysis (glucose, glucose‐6‐P, fructose‐6‐P) (*P* < 0.01), tricarboxylic acid cycle (succinyl‐CoA, malate) (*P* < 0.001), and gluconeogenesis‐related metabolite, alanine (*P* < 0.01), are significantly upregulated by DR. The notably (*P* < 0.01) down‐modulated muscle growth (PGF_2α_) and regeneration (PGE_2_) stimulation metabolite and the increased consumption of glycogen in muscle and liver may be related to the significantly (*P* < 0.01) lower body weight and muscle mass by DR. The downregulated oxidative stress, pro‐inflammatory mediators, and upregulated anti‐inflammatory metabolites resulted in a lower energy expenditure, which contributed to enhanced muscle quality together with upregulated energy production pathways by DR. The improved muscle quality may explain why grip strength is maintained and motor coordination and learning performance are improved by DR in *Ercc1* ^*∆/−*^ and *Xpg* ^*−/−*^ mice.

**Conclusions:**

This study provides fundamental supporting information on biomarkers and pathways related to the attenuation of sarcopenia, which might facilitate its diagnosis, prevention, and clinical therapy.

## Introduction

Sarcopenia, the age‐related decline of muscle mass and strength, constitutes a major health problem, and is one of the main causes of loss of independence in the elderly.^1^ Muscle mass usually starts to decline from about 30 years of age, with on average 40% of muscle mass lost by the age of 80 years.^2^ Worldwide, 11–50% of those aged 80 or above suffer from sarcopenia,^3^ and this number is increasing with the rise of average life expectancy. These developments impose severe pressure on healthcare resources and constitute an enormous socio‐economic burden.^4^ Medication for sarcopenia is unfortunately lacking,^5^ and the molecular mechanisms underlying sarcopenia are still poorly understood.

Previous studies revealed that oxidative stress and inflammatory mediators (i.e., TNF‐α and GSSG),^6^, ^7^, ^8^ and lipid peroxidation products – polyunsaturated fatty acids (PUFAs) that regulate a diverse set of inflammatory processes (i.e., 15‐HEPE, 13‐HOTrE, 17‐HDOHE, PGE_2_, PGD_2_, XTB_2_, 9‐HODE, and 11‐HETE),^8^, ^9^, ^10^, ^11^ play important roles in sarcopenia, and are associated with the loss of muscle mass^12^ and grip strength,^13^ an indicator of muscle strength, in mice and human. Energy production related molecules and amino acids were also reported to be associated with muscle contractile function, and other age‐associated diseases in numerous species.^14^, ^15^ Several lifestyle interventions can affect health and longevity, including dietary intake and exercise.^16^ Among those, caloric‐ or dietary restriction (CR/DR; reduced food intake without malnutrition) is currently recognized as effective intervention for extending lifespan and retarding age‐related diseases in numerous species, including mammals^17^, ^18^, ^19^ and humans,^20^ and references therein]. Besides the indications of DR attenuating sarcopenia, muscle atrophy, and motor neuron loss,^18^, ^21^, ^22^ the fundamental mechanism of how DR attenuates sarcopenia still needs to be systematically investigated to identify potential biomarkers for monitoring diagnosis, prevention, and treatment of sarcopenia.

Aging and age‐related diseases can be influenced by many factors. One of the main causal hallmarks of aging is time‐dependent accumulation of DNA damage, also known as genomic instability.^23^, ^24^ *ERCC1* is a critical DNA binding subunit in the *ERCC1–XPF* protein complex, which forms an active endonuclease in DNA damage repair and cuts DNA specifically at junctions between single‐ and double‐stranded DNA, ~20 nt 5′ to the lesion. The *XPG* gene codes for a structure‐specific endonuclease that cleaves damaged DNA ~5 nt 3′ to the site of the lesion.^25^, ^26^ Mice deficient in the DNA excision‐repair genes *Ercc1* (*Ercc1* ^*∆/*−^) or *Xpg* (*Xpg* ^*−/−*^) cannot properly repair multiple types of DNA lesions leading to an accelerated accumulation of persisting DNA damage, which stall elongating RNA polymerases causing transcription stress, leading to reduced and gene‐length dependent transcriptional decline particularly in post‐mitotic tissues.^17^, ^27^ Importantly, genome‐wide transcription stress causing lowered and skewed transcriptional output has subsequently also been discovered in normal aging in mice and to be widely evolutionary conserved from worms to human,^27^ demonstrating the value of progeroid DNA‐repair‐deficient mouse mutants for understanding the natural aging and aging‐related diseases.^23^ Because transcription is at the basis of all cellular processes, and transcription stress triggers a complex DNA damage response,^25^ it has numerous secondary and tertiary consequences. This explains the wide range of pathological, physiological and behavioural features associated with the multi‐morbidity of natural aging and the accelerated aging in repair mouse mutants and corresponding human syndromes, including progressive neurodegeneration (dementia, ataxia, hearing and vision loss); osteoporosis; liver, kidney, vascular and haematological aging; and so on.^17^, ^26^, ^27^, ^28^, ^29^ Progeroid *Ercc1* ^*∆/*−^ mice were previously successfully used for studying and modulating many features of aging^17^ including sarcopenia.^30^

Metabolite profiling is an important tool to link genotype and phenotype, and hence constitutes a powerful approach for the investigation of complex diseases and identification of diseases' biomarkers,^31^ such as oxidative stress and inflammatory markers (e.g., several signalling lipids), and energy status (ATP, ADP, glucose, etc.). Therefore, in this paper, metabolomics was utilized to systematically investigate metabolites related to oxidative stress, (pro‐ and anti‐) inflammatory status, and energy production and consumption in wild type, *Ercc1* ^*∆/*−^ and *Xpg* ^*−/−*^ mouse models under *ad libitum* (AL) fed and DR conditions with the aim to provide reference information for the potential biomarkers of sarcopenia attenuation, and facilitate the diagnosis, prevention, and treatment of sarcopenia.

## Methods

### Mouse models

*Ercc1* ^∆/−^ mice were obtained by crossing *Ercc1* ^∆/+^ with *Ercc1* ^+/−^ mice, which have been previously described.^32^ *Xpg* ^−/−^ mice have been generated and characterized previously^33^ by crossing *Xpg* ^+/−^ with *Xpg* ^+/−^ mice. Wild‐type (WT) F1 littermates (+/+ genotypes from *Ercc1* ^∆/−^ and *Xpg* ^−/−^ breedings) were used as controls. Only male mice were used in this paper to minimize the influence of gender on DR study. All of the animals used were in full accordance with European legislation, and detailed in the supporting information.

### Dietary intervention and housing conditions

Mice were housed in individual ventilated cages under specific pathogen‐free conditions (20–22°C, 12–12 h light–dark cycle with light phase adjusted to between 24:00 and 12:00 h) and provided food and water *ad libitum* (AL). Because the *Ercc1* ^∆/−^ and *Xpg* ^−/−^ mice were smaller, food was administered within the cages, and water bottles with long nozzles were used from around 2 weeks of age. Mice were weighed, visually inspected weekly, and scored blindly for gross morphological and motor abnormalities weekly. All efforts were made to ameliorate the suffering of the animals. Animals were bred and maintained on AIN93G synthetic pellets (Research Diet Services B. V.; gross energy content 4.9 kcal/g dry mass, digestible energy 3.97 kcal/g). Animals were divided randomly over all groups to prevent selection bias. Dietary restriction (DR) was applied as published before.^17^ On average, *Ercc1* ^∆/−^ and *Xpg* ^−/−^ mice ate 2.3 g food per day. DR was initiated gradually starting from 7 weeks of age with 10% food reduction (2.1 g/day), when animals reached almost‐maximum bodyweight and development was completed, and was increased weekly by 10%, until it reached 30% DR (1.6 g/day) from 9 weeks of age until 16 weeks. WT mice ate on average 3.0 g food per day, resulting in 2.1 g/day for 30% DR. Food was given to the animals just before the start of the dark (active) period, Zeitgeber Time (ZT) 12:00, to minimize disturbance of the biological clock. All mice were sacrificed and muscle samples were collected within a time frame of 1–3.5 h after feeding in their active period, between ZT 13:00 and 15:30, to avoid nutritional or circadian fluctuations of metabolites due to differences in the time after feeding and the biological clock.

### Behavioural analyses

Motor coordination performance was assessed by measuring the average time spent on an (2–40 rpm) accelerating rotarod (Ugo Basile) and detailed in Supplementary Information.

### Muscle tissue isolation and blood glucose measurement

Mice were killed by CO_2_ asphyxiation and a large piece of quadriceps femoris muscle (Quad) was dissected and rapidly frozen in liquid nitrogen, stored in −80°C, then lyophilized for 24 h, and weighed before analysis. Blood glucose was determined with a Freestyle mini blood glucose meter just before mice collection.

### Metabolomic analysis preparation

The chemicals, solvents, internal standards (ISTDs) used for muscle tissue extraction as well as the muscle tissue extraction method were described in He et al.^34^ and detailed in the supporting information.

### Metabolite analysis

A portion of extracted mouse muscle tissue for a pre‐experiment was used as quality control (QC) samples. A QC sample was injected once each six to eight samples to evaluate and correct for changes in sensitivity of the instruments.

### Lipid metabolite analysis

The lipid metabolites were analysed by a validated ultra‐performance liquid chromatography tandem mass spectrometry (UPLC‐MS/MS) method.^35^ Each sample was measured with two complementary reverse phase methods using mobile phases with different pH, method details in the supporting information.

### Analysis of energy‐production‐related metabolites

The energy‐production‐related metabolites were analysed by a hydrophilic interaction liquid chromatography (HILIC) mass spectrometry platform,^36^ the method details are described in the supporting information.

### Data analysis

For each metabolite analysed by UPLC‐MS(/MS), the response ratio was obtained by Equation (1), normalized by the muscle tissue dry weight, and corrected by the QC samples:

(1) $$\text{Response ratio}=\;\frac{\text{Peak area of the target metabolite}\;}{\text{Peak area of the assigned ISTD}}÷\text{muscle tissue}\;\mathrm{dry}\;\text{weight}$$

For metabolites that can be measured by multiple platforms, that is, some fatty acids can be determined with both low and high pH lipid method, the results with smaller QC RSD were utilized (Table S1).

The fold change of metabolites regulated by DR was calculated by the Equation (2):

(2) $$\text{Fold}\;\text{change}=\frac{\text{Response ratio of}\;\mathrm{DR}}{\text{Response ratio of}\;\mathrm{AL}}$$

RStudio (Version 1.4.1106) and R (Version 4.0.5) were used for data statistical analysis, and figure plotting.

## Results

### Differential effects of dietary restriction on mouse body weight, muscle mass and muscle functioning in wild‐type and progeroid DNA‐repair‐deficient mutant mice

**Figure 1:**
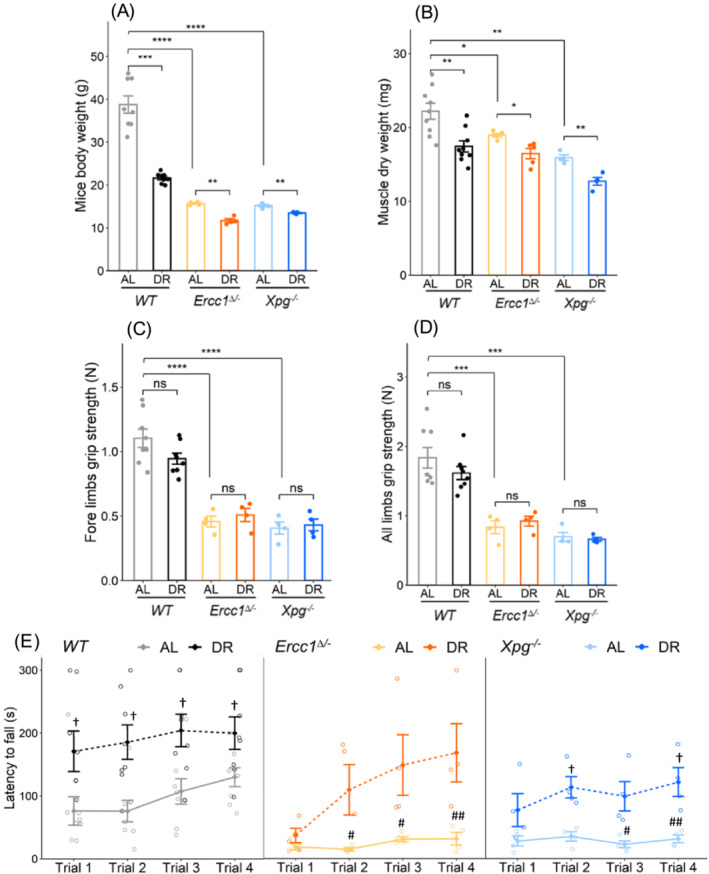
The effects of dietary restriction on body weight, muscle dry weight, grip strength, and motor coordination in *WT* (*n* = 8 or 9), *Ercc1* ^*∆/−*^ (*n* = 4 or 5), and *Xpg* ^*−/−*^ (*n* = 4) mice with only male and age 14/16 weeks. *P < 0.05, **P < 0.01, ***P < 0.001, ****P < 0.0001, *t* test with false discovery rate (FDR) correction. †*P* < 0.05, *t* test with FDR correction between AL and DR in each type of mice. # *P* < 0.05, ## *P* < 0.01, *t* test with FDR correction between *WT* and mutated (*Ercc1* ^*∆/−*^ *or Xpg* ^*−/−*^) animals under AL conditions in each trial. (A) Mouse body weight, (B) quadriceps femoris muscle (Quad) dry weight, (C) fore limbs grip strength, (D) all limbs grip strength, (E) motor learning and coordination performance. (Trial 1, 2, 3 and 4 means the 1st, 2nd, 3rd and 4th trial, respectively). The dots represent the value of each replicate, and the error bars represent standard error.

We previously have shown that interventions like DR can yield significant benefits to health and lifespan in progeroid DNA‐repair‐deficient mice.^17^, ^30^, ^37^ Here, we first investigated the effects of DR (starting at the age of 7 weeks) on sarcopenia by monitoring mouse body weight, muscle mass, grip strength, and accelerating rotarod performance in 16‐week‐old *Ercc1* ^*∆/−*^ mice and 14‐week‐old *Xpg* ^*−/−*^ mice, with corresponding WT littermates. These ages correspond to approximately 75% of their maximum lifespan and at which both mouse models display progressive age‐related pathologies across many organs, including severe muscle wasting.^17^, ^30^ Under AL feeding conditions, body weight (*P* < 0.0001, Figure 1A) and muscle mass (*P* < 0.05, Figure 1B) of *Ercc1* ^*∆/−*^ and *Xpg* ^*−/−*^ mice were substantially lower compared to WT littermates, and all were significantly (*P* < 0.05) decreased by DR, ranging from 11% to 44% for body weight (Figure 1A) and from 13% to 20% for muscle mass (Figure 1B), consistent with previous data.^22^ *Ercc1* ^*∆/−*^ and *Xpg* ^*−/−*^ mice showed a significantly (*P* < 0.001) reduced muscle strength (Figure 1C,D), which might be the result of their muscle mass waste (Figure 1B).^30^ However, DR did not affect their fore limbs and all limbs grip strength even with significantly decreased muscle mass (Figure 1C,D). This indicates improved muscle quality, which is defined as the amount of strength per unit of muscle mass and is a critical biomarker of muscle health.^38^ These results are consistent with the recent observation in human muscle.^20^ One of the detrimental effects of sarcopenia in the elderly is loss of motor coordination.^39^ To further investigate the effects of DR on muscle quality, four trials of accelerating rotarod performance were conducted. In line with their accelerated aged phenotype, AL‐fed *Ercc1* ^*∆/−*^ and *Xpg* ^*−/−*^ mice showed a significantly (*P* < 0.05) reduced motor coordination compared to WT mice (Figure 1E), consistent with the adverse effect of sarcopenia on motor coordination, and fully in line with previous observations.^30^, ^39^ DR strongly (*P* < 0.05) improved the motor coordination and learning (Figure 1E). In particular, *Ercc1* ^*∆/−*^ and *Xpg* ^*−/−*^ mice were able to equally control motor coordination as normal WT mice in the fourth trial, further (partially) demonstrating the improved muscle quality by DR. *Ercc1* ^*∆/−*^ DR mice were poor in rotarod performance at the first trial but systematically improved. These mice have a longer lifespan and seem to profit more from DR than the *Xpg* ^*−/−*^ mutants,^17^ which are already more advanced in overall aging compared to *Ercc1* ^*∆/−*^ at the start of DR. This indicates that the improved neurological performance in *Ercc1* ^*∆/−*^ mice also noted before,^17^ which may also partially contribute to the improved motor coordination and learning performance.

### The effects of accelerated aging on oxidative‐stress‐related, pro‐inflammatory, anti‐inflammatory, and energy‐related metabolites

**Figure 2:**
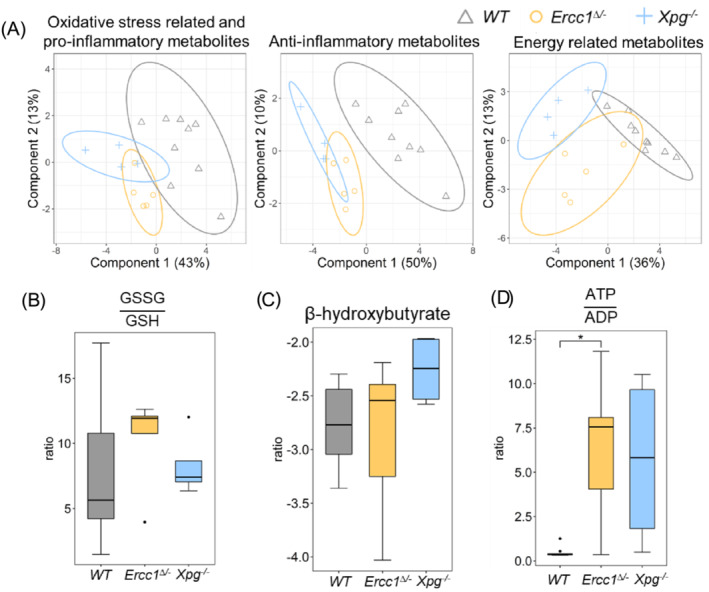
(A) The partial least squares‐discriminant analysis (PLS‐DA) score plot for the effect of accelerated aging on oxidative‐stress‐related, pro‐inflammatory, anti‐inflammatory, and energy‐related metabolites in AL mice; the effects of accelerated aging on the representative indicators of (B) oxidative stress and pro‐inflammation, (C) anti‐inflammation, and (D) energy status. *WT* (*n* = 9), *Ercc1* ^*∆/−*^ (*n* = 5), and *Xpg* ^*−/−*^ (*n* = 4). *P < 0.05, *t* test with FDR correction.

The significantly decreased grip strength, motor coordination and learning performance in AL progeroid mice compared to AL‐WT mice (Figure 1) may be related to altered muscle function and quality. Hence, muscle specimens of the quadriceps femoris muscle (Quad) were profiled using recently developed metabolomics methods for the detection of signalling lipids and polar metabolites^34^ and the identified molecules related to muscle function and quality, that is, oxidative stress, pro‐ and anti‐inflammatory markers, and energy status, analysed by Partial Least Squares Discriminant Analysis (PLS‐DA). Separation between WT and *Ercc1* ^*∆/−*^ or *Xpg* ^*−/−*^ mice was observed, especially for the anti‐inflammatory metabolites (Figure 2A), indicating large alterations of accelerated aging in these metabolites.

Three indicators of oxidative stress, anti‐inflammation, and energy status were used to profile the effects of accelerated aging. The ratio of oxidized glutathione (GSSG) to reduced glutathione (GSH) is an important marker of oxidative stress.^40^ The trend for increased GSSG/GSH ratios in *Ercc1* ^*∆/−*^ (and *Xpg* ^*−/−*^) animals compared to WT mice, which did not reach statistical significance (Figure 2B) might be related to the slightly improved oxidation defence, suggested by the increase of β‐hydroxybutyrate in *Xpg* ^*−/−*^ mice (Figure 2C), an important indicator of anti‐aging effects [S1]. The ratio of ATP to ADP in *Ercc1* ^*∆/−*^ and *Xpg* ^*−/−*^ mice (Figure 2D) was significantly (*P* < 0.05) elevated comparing with the WT mice.

### The effects of dietary restriction on metabolites and pathways in pro‐inflammation, oxidative stress, and muscle growth stimulation

**Figure 3:**
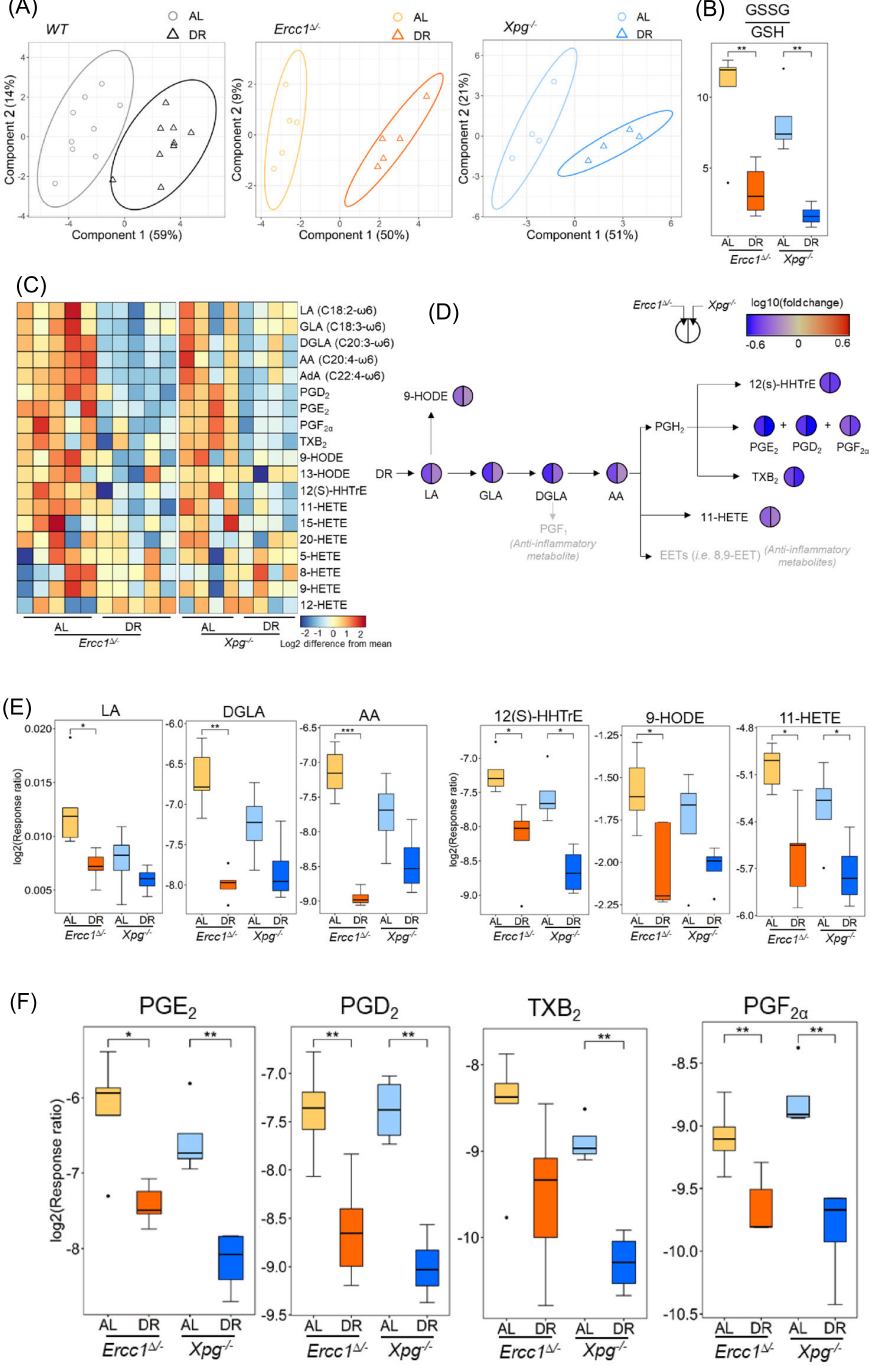
Footprint of the effects of DR on pro‐inflammatory and oxidative‐stress‐related pathways and metabolites for sarcopenia in *WT* (*n* = 9), *Ercc1* ^*∆/−*^ (*n* = 5), and *Xpg* ^*−/−*^ (*n* = 4) mice. (A) The PLS‐DA score plot for the effect of DR on pro‐inflammatory and oxidative‐stress‐related metabolites. (B) The effects of DR on oxidative stress indicator, the ratio of GSSG to GSH in *Ercc1* ^*∆/−*^ and *Xpg* ^*−/−*^ mouse muscle (we focused here and in the other analyses on the *Ercc1* ^*∆/−*^ and *Xpg* ^*−/−*^ mice because of the overall strong effect of DR in these mutants). (C) Heatmap representation of the pro‐inflammatory and oxidative‐stress‐related metabolites between AL and DR in *Ercc1* ^*∆/−*^ and *Xpg* ^*−/−*^ mouse muscle (each column represents one mouse). (D) The fold change of metabolites significantly (*P* < 0.05) regulated by DR in the pro‐inflammatory pathways (*t* test with FDR correction). (E, F) The effects of DR on (E) ω6 polyunsaturated fatty acids and ROS‐stimulated metabolites, (F) pro‐inflammatory, muscle growth and regeneration stimulation metabolites in *Ercc1* ^*∆/−*^ and *Xpg* ^*−/−*^ mouse muscle. *P < 0.05, **P < 0.01, ***P < 0.001, *t* test with FDR correction.

To identify the fundamental molecular changes of DR on inflammation and oxidative stress in sarcopenia, lipidomics analysis was performed on muscle of *Ercc1* ^*∆/−*^, *Xpg* ^*−/−*^ and WT mice by measuring pro‐inflammatory and oxidative‐stress‐related metabolites, and lipid peroxidation products, that is, ω6 polyunsaturated fatty acids (PUFAs), prostaglandin (PG) series‐2, thromboxanes (TX) series‐2, hydroxyoctadecadienoic acids (HODEs, 12(S)‐hydroxyheptadecatrienoic acid (HHTrE), and hydroxyeicosatetraenoic acids (HETEs).^8^, ^11^ The resulting metabolic profiles of PLS‐DA showed clear differences between AL and DR in *Ercc1* ^*∆/−*^ and *Xpg* ^*−/−*^ mice (Figure 3A), indicating that DR exerts large effects on pro‐inflammatory and oxidative‐stress‐related metabolites in the progeroid mice muscle. This is consistent with the very strong anti‐aging effect of DR in the progeroid repair mutants.^17^ In view of the high impact of DR in the *Ercc1* ^*∆/−*^ and *Xpg* ^*−/−*^ mice, we decided to investigate the effect of DR in more detail. Significantly (*P* < 0.01) decreased GSSG/GSH in *Ercc1* ^*∆/−*^ and *Xpg* ^*−/−*^ mice demonstrated reduced oxidative stress by DR in progeroid mice (Figure 3B). The heatmap profiles of the metabolites (Table S3) related to oxidative stress and pro‐inflammation clearly show an overall reduction by DR in both *Ercc1* ^*∆/−*^ and *Xpg* ^*−/−*^ mice (Figure 3C). To better visualize the DR effects, the fold changes (Equation 2) of significantly (*P* < 0.05) regulated metabolites in the schematic pathways related to pro‐inflammatory and oxidative stress were profiled (Figure 3D). The linoleic acid (LA) and arachidonic acid (AA) pathways and metabolites (Figure 3E,F) related to pro‐inflammation and oxidative stress, that is, dihomo‐γ‐linolenic acid (DGLA), AA, 12(S)‐HHTrE,^11^ 9‐HODE,^8^ 11‐HETE,^8^, ^10^ PGE_2_, PGD_2_, PGF_2α_,^8^ and TXB_2_,^11^ and muscle growth (PGF_2α_) and regeneration (PGE_2_) stimulators [S2–S4] were all significantly (*P* < 0.05) and consistently downregulated by DR in both models.

### The effects of dietary restriction on anti‐inflammatory metabolites and pathways

**Figure 4:**
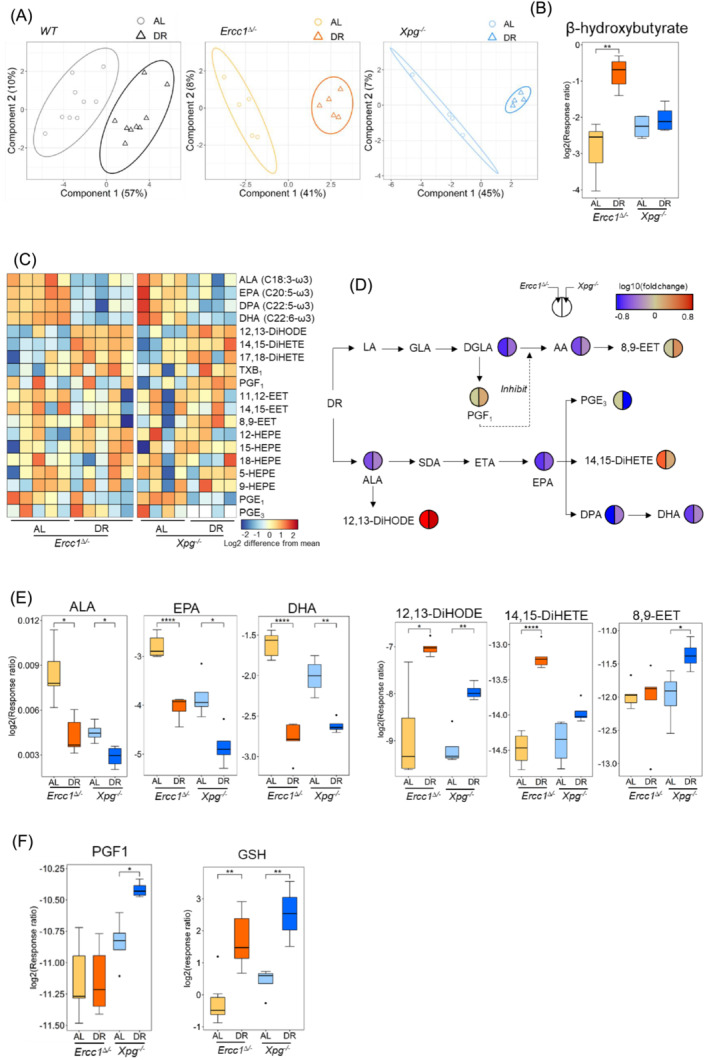
Footprint of the effects of DR on anti‐inflammatory metabolites and pathways for sarcopenia in *WT* (*n* = 9), *Ercc1* ^*∆/−*^ (*n* = 5), and *Xpg* ^*−/−*^ (*n* = 4) mice. (A) The PLS‐DA score plot for the effect of DR on anti‐inflammatory metabolites. (B) The effects of DR on anti‐aging indicator (β‐hydroxybutyrate) in *Ercc1* ^*∆/−*^ and *Xpg* ^*−/−*^ mouse muscle. (C) Heatmap profile of the anti‐inflammatory metabolites between AL and DR in *Ercc1* ^*∆/−*^ and *Xpg* ^*−/−*^ mouse muscle (background colour was used for the non‐detected metabolites, and each column represents one mouse). (D) The fold change of metabolites significantly (*P* < 0.05) regulated by DR in the anti‐inflammatory pathways (*t* test with FDR correction). (E, F) The effects of DR on (E) ω3 polyunsaturated fatty acids and anti‐inflammatory metabolites, (F) metabolites in LA pathway to inhibit the AA generation and GSH in *Ercc1* ^*∆/−*^ and *Xpg* ^*−/−*^ mouse muscle. *P < 0.05, **P < 0.01, ***P < 0.001, ****P < 0.0001, *t* test with FDR correction.

The altered concentrations of oxidative stress markers and pro‐inflammation signalling lipids associated with DR may be not only due to the reduced levels of reactive oxygen species (ROS) or reactive nitrogen species (RNS), but also caused by the modulation of anti‐inflammatory cytokines [S5], which presented protective antioxidant defences in *Ercc1* ^*∆/−*^ and *Xpg* ^*−/−*^ mice [S6], such as eicosapentaenoic acid (EPA), PG series‐1 and series‐3, TX series‐1, epoxyeicosatrienoic acids (EETs), dihydroxy‐eicosatetraenoic acids (DiHETEs), and dihydroxy octadecadienoic acids (DiHODEs) [S7–S11]. PLS‐DA profiles of these anti‐inflammatory metabolites clearly segregated AL and DR groups in *Ercc1* ^*∆/−*^ and *Xpg* ^*−/−*^ mice (Figure 4A), demonstrating the more potent robustness in regulation of anti‐inflammatory mediators by DR in the muscle of progeroid mice. β‐Hydroxybutyrate is reported as an important marker of the anti‐aging effects of DR and fasting [S1]. Significantly (*P* < 0.001) increased β‐hydroxybutyrate indicated improved anti‐inflammation by DR in progeroid *Ercc1* ^*∆/−*^ mice (Figure 4B). The heatmap profile of analysed anti‐inflammation metabolites (Table S4) in *Ercc1* ^*∆/−*^ and *Xpg* ^*−/−*^ muscle showed an obvious downregulation of ω3 PUFAs (i.e., α‐Linolenic acid (ALA), EPA, docosapentaenoic acid (DPA), and docosahexaenoic acid (DHA)), and several clearly upregulated anti‐inflammatory mediators (i.e., 14,15‐DiHETE, TXB_1_, PGF_1_, and 12,13‐DiHODE) by DR in the progeroid sarcopenic mice (Figure 4C). Using a cut‐off *P*‐value of <0.05 for the fold change (Equation 2) of metabolites (Figure 4D), we clearly observed significantly down‐regulated (i.e., ALA, EPA, DPA, DHA, and PGE_3_), and up‐regulated mediators (i.e., PGF_1_, 8,9‐EET, 12,13‐DiHODE, 14,15‐DiHETE, and GSH) by DR in the anti‐inflammation‐related pathways in *Ercc1* ^*∆/−*^ and *Xpg* ^*−/−*^ mice (Figure 4E,F).

### The effects of dietary restriction on energy production related metabolites and pathways

**Figure 5:**
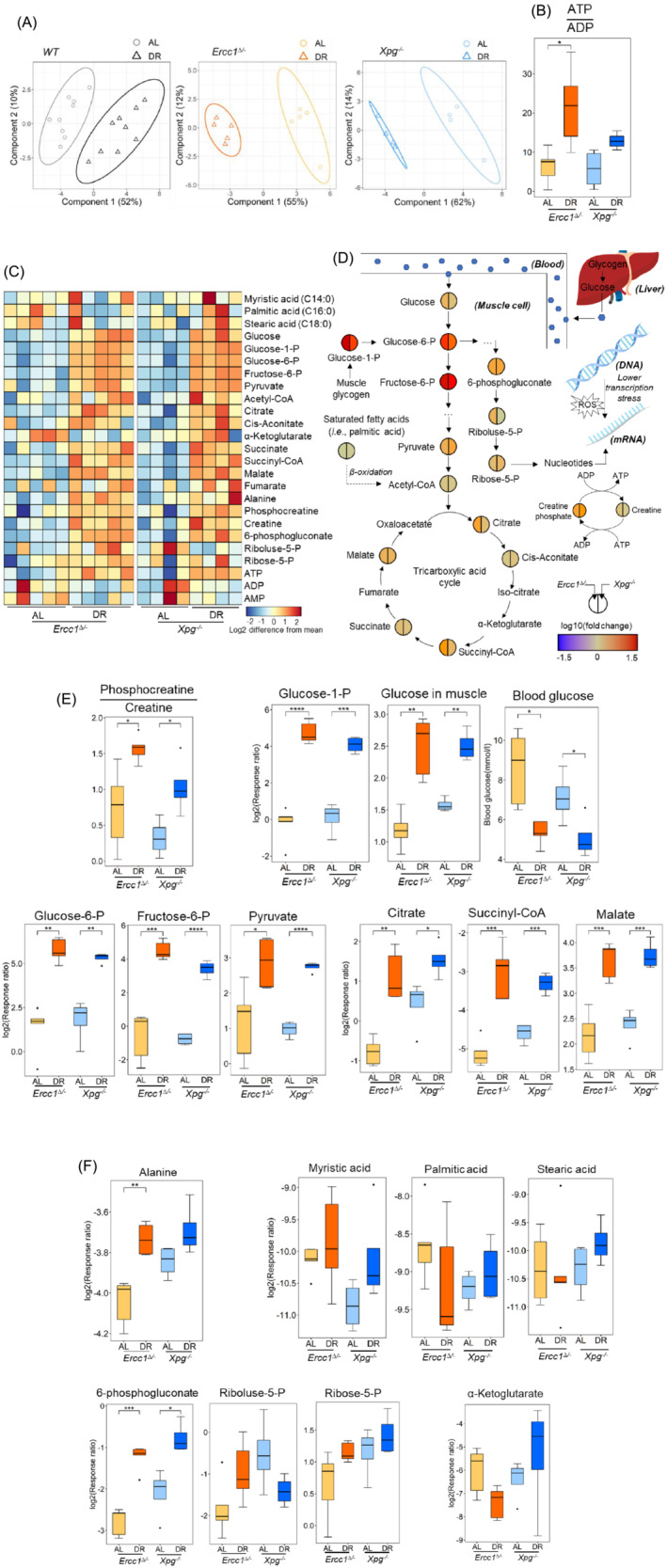
Footprint of the effects of DR on energy‐generation‐related metabolites and pathways for sarcopenia in *WT* (*n* = 9), *Ercc1* ^*∆/−*^ (*n* = 5), and *Xpg* ^*−/−*^ (*n* = 4) mice. (A) The PLS‐DA score plot for the effect of DR on energy‐generation‐related metabolites. (B) The effects of DR on the ratio of ATP to ADP in *Ercc1* ^*∆/−*^ and *Xpg* ^*−/−*^ mouse muscle. (C) Heatmap profile of the energy‐generation‐related metabolites between AL and DR in *Ercc1* ^*∆/−*^ and *Xpg* ^*−/−*^ mouse muscle (each column represents one mouse). (D) The fold change of metabolites significantly (*P* < 0.05) regulated in the energy‐generation‐related pathways (*t* test with FDR correction). (E, F) The effects of DR on (E) phosphocreatine to creatine, energy substrates (i.e., glucose in muscle and blood, and glucose‐1‐phosphate in muscle), metabolites in glycolysis, and metabolites in tricarboxylic acid cycle; (F) metabolite that stimulating gluconeogenesis, saturated fatty acids, metabolites in pentose phosphate pathway (PPP), and α‐ketoglutarate in *Ercc1* ^*∆/−*^ and *Xpg* ^*−/−*^ mouse muscle. *P < 0.05, **P < 0.01, ***P < 0.001, ****P < 0.0001, *t* test with FDR correction.

Energy production in muscle tissue is highly related to grip strength and muscle function [S12]. To determine the DR effects on muscle energy generation in sarcopenia, the related metabolites of glycolysis, tricarboxylic acid (TCA) cycle, gluconeogenesis, and saturated fatty acids (SFAs) lipolysis, were all analysed. A clear segregation between AL and DR was observed in *Ercc1* ^*∆/−*^ and *Xpg* ^*−/−*^ mice (Figure 5A), indicating high modulation of energy‐production‐related metabolites by DR in the muscle of progeroid mice. Significantly (*P* < 0.05) increased ATP/ADP ratio demonstrated improved energy status by DR in *Ercc1* ^*∆/−*^ and *Xpg* ^*−/−*^ mice (Figure 5B). The influence of fasting on the ATP/ADP ratio and energy status was minimized by sacrificing mice and collecting muscle samples 1–3.5 h after feeding, when mice had just finished or were finishing their food intake. Phosphocreatine is considered as the “energy pool” in muscle cells, and is preferentially consumed by generating creatine in case of an insufficient energy supply [S13]. A heatmap profile of all analysed energy‐production and storage‐related metabolites (Table S5) showed that most of the analytes were highly upregulated by DR (Figure 5C). To profile the DR effects, the fold change (Equation 2) of significantly (*P* < 0.05) modulated metabolites in the main energy generation pathways, that is, glycolysis and TCA, and metabolites related to pentose phosphate pathway (PPP), gluconeogenesis, SFAs lipolysis, and phosphocreatine were shown in their pathways in Figure 5D. The significantly increased fold change of key metabolites in glycolysis and TCA cycle indicated upregulation of energy production and storage pathways by DR in *Ercc1* ^*∆/−*^ and *Xpg* ^*−/−*^ mice.

Phosphocreatine is generated from creatine by receiving a high‐energy phosphate group split from ATP [S13], therefore higher ratio of phosphocreatine to creatine represents a higher energy status of muscle tissue. The significantly (*P* < 0.05) increased ratio of phosphocreatine to creatine further revealed that the energy status of muscle was notably improved by DR in *Ercc1* ^*∆/−*^ and *Xpg* ^*−/−*^ mice (Figure 5E). Glycogen, primarily in liver and muscle cells, is an important source to maintain energy balance. Fasting and DR can stimulate the transformation of liver and muscle glycogen to glucose and glucose‐1‐phosphate (glucose‐1‐P), respectively, to feed into glycolysis for energy production. The significantly (*P* < 0.001) increased glucose‐1‐P in muscle tissue demonstrated the high consumption of muscle glycogen during DR (Figure 5E). Muscle consumes nearly 80% of the body's glucose content [S14, S15]. The significantly (*P* < 0.01) upregulated glucose levels in muscle indicated that glucose is transported and absorbed from blood to muscle tissue because of DR (Figure 5E). This is consistent with the decreased blood glucose content by DR in Figure 5E and Vermeij et al.^17^ Notably (*P* < 0.05) increased glycose‐6‐phosphate (glycose‐6‐P), fructose‐6‐phosphate (fructose‐6‐P), and pyruvate by DR revealed significantly upregulated glycolysis by DR in progeroid mice (Figure 5E). Similarly, the notable (*P* < 0.05) increased citrate, succinyl‐CoA, and malate indicated a significant upregulation of TCA by DR in progeroid mice (Figure 5E).

Alanine was reported to be able to enhance gluconeogenesis in starvation [S16]. Significantly (*P* < 0.01) increased alanine also revealed upregulated gluconeogenesis by DR in muscle of progeroid mice (Figure 5F). SFAs are another potential energy source during DR and three important SFAs, that is, myristic acid, palmitic acid, and stearic acid, were determined in *Ercc1* ^*∆/−*^ and *Xpg* ^*−/−*^ mouse muscle specimens. The results showed that all these SFAs were unaffected by DR (Figure 5F), indicating that DR did not induce lipolysis of SFAs in progeroid mice. Hence, the increased energy should originate from glycolysis, TCA, and gluconeogenesis from glycogen.

The significant (*P* < 0.05) increase of 6‐phosphogluconate by DR may be due to the elevated level of its precursor glucose‐6‐P (Figure 5F). Ribose 5‐phosphate (ribose‐5‐P) is the source for the synthesis of nucleotides. The unaffected ribose‐5‐P and ribulose‐5‐phosphate (ribulose 5‐P) levels by DR (Figure 5F) indicated that these precursors of nucleotides synthesis were not accumulating, consistent with the general suppression of growth and enhanced salvage pathways by DR.

## Discussion

**Figure 6:**
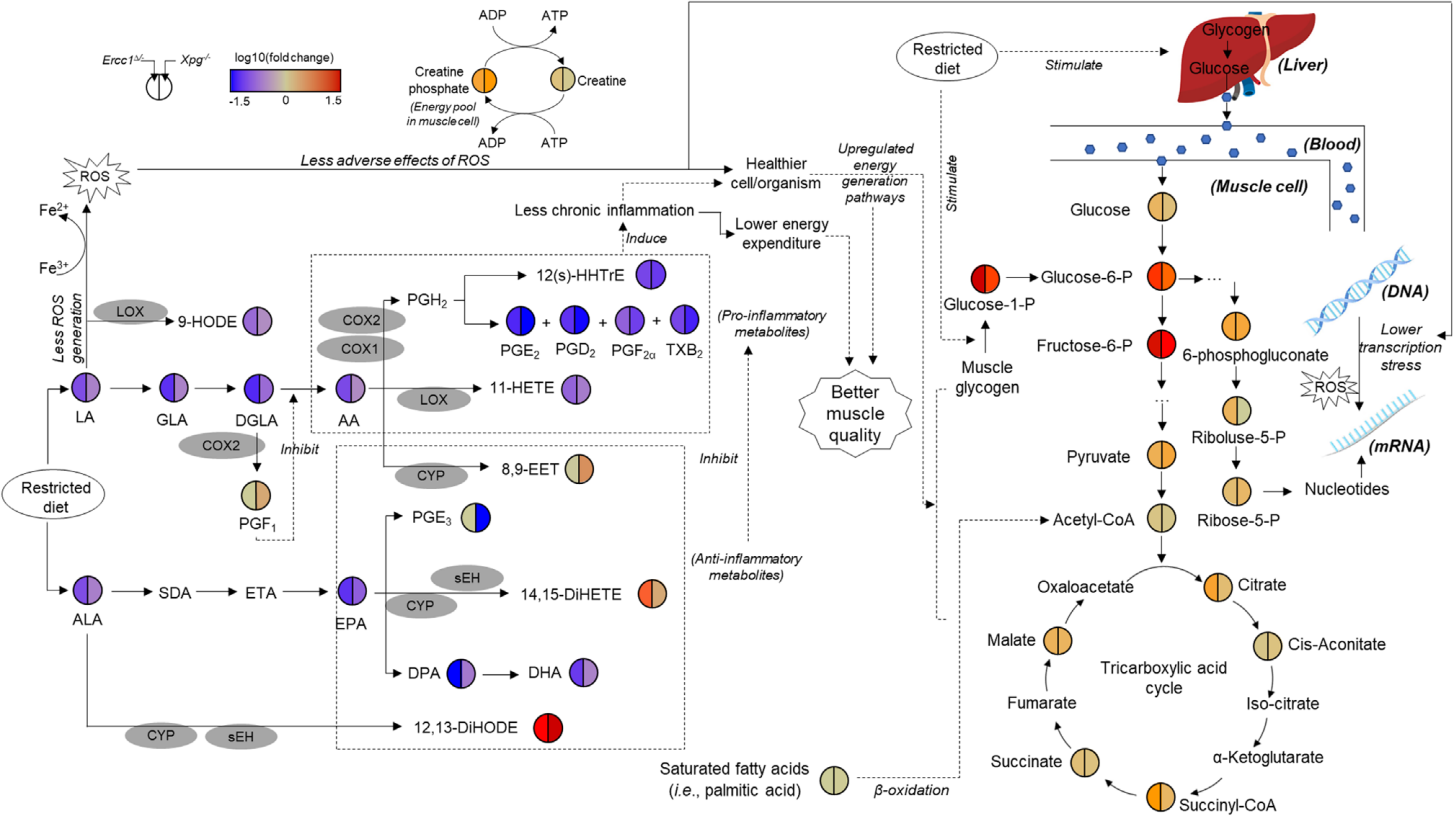
Overview of the metabolites and pathways regulated by DR to improve muscle quality and function in sarcopenia in DNA‐repair‐deficient prematurely aging mouse mutants.

Our data showed that DR significantly decreased body weight and muscle mass, and improved motor learning and coordination in DNA‐repair‐deficient progeroid mice, consistent with studies in naturally aged mice [S17]. Sarcopenia is a result of several impaired pathways in the aging process and DNA damage is one part of it. Analysis of key energy metabolites showed that DR‐treated animals exhibited enhanced conversion of liver and muscle glycogen to glucose and glucose‐1‐P, respectively, to feed into glycolysis in muscle, and simultaneously upregulated TCA, other pathways of gluconeogenesis, and energy storage in phosphocreatine (Figure 6). The consumption of liver and muscle glycogen may in part relate to the decreased mouse body weight and muscle mass (Figure 1A,B). This might at least in part be due to the short time (~1–3.5 h) between feeding and harvesting of the animals, and the standardized protocol for feeding DR mice (ZT 12:00), which minimized the effect of biological clock on DR mice and the narrowed time window of harvesting. Xie et al. also reported that caloric restriction increases energy production and supply for muscle exercise, and significantly improves muscle work efficiency [S15]. The further increased energy status by DR, that is, ATP/ADP ratio (Figure 5B), may be due to the upregulation of the two major energy generation pathways, glycolysis and TCA, which is distinct from the reduced consumption of ATP by the increased transcription blocking DNA lesions (TBLs) and transcription stress in AL‐fed *Ercc1* ^*∆/−*^ and *Xpg* ^*−/−*^ mice (Figure 2D) [S6]. The stochastic accumulation of TBLs affect many genes, including nuclear encoded mitochondrial genes, which contribute to the mitochondrial structure deficits noted before in *Ercc1* ^*∆/−*^ mice muscle.^30^ The improved muscle energy status, that is, increased ATP/ADP and phosphocreatine/creatine ratio may contribute to maintained grip strength and improved motor coordination by DR (Figure 1).

The improved energy metabolism by DR may be enabled by the lower DNA‐damage‐induced transcription stress,^17^, ^27^ leading to improved gene expression output. Conversely, the metabolic redesign brought about by DR may lower the endogenously generated DNA damage load and thereby diminish the transcription stress, caused by transcription‐stalling DNA lesions [17, S6]. Importantly, the occurrence of DNA‐damage‐induced transcription stress in natural aging^27^ supports a similar scenario in physiological situations of age‐associated sarcopenia. Interestingly, α‐ketoglutarate (aKG), which was elevated locally in *Xpg* ^−/−^ muscle upon DR (Figure 5F), has been recently implicated in extension of lifespan and compression of morbidity [S18], opening opportunities for further research for assessing and modulating aKG levels systemically for counteracting accelerated aging and sarcopenia.^30^

Poly‐unsaturated fatty acids (PUFAs) are among the most oxygen‐sensitive components in nature, and were reported as the main generator of superoxide by the reaction of lipoxygenases catalysed by metal ions, in particular iron ions [S19]. LA and ALA are the two essential fatty acids for animals and human, must be obtained from food. LA is the main PUFA in most diets, typically consumed in 5‐ to 20‐fold greater amounts than ALA [S20], and is present in around 8.4‐fold larger amounts than ALA in synthetic AIN93G chow, the food provided to the mice in this study [S21]. DR significantly reduced the supply of these two essential PUFAs, LA and ALA, leading to their downregulation in *Ercc1* ^*∆/−*^ and *Xpg* ^*−/−*^ mouse muscle. DR also contributes to the lower iron accumulation in skeletal muscle [S22]. Both the decreased LA content and reduced iron accumulation may at least in part account for the lower level of oxidative stress in progeroid mouse muscle (Figures 3B and 6), as apparent from the downregulation of GSSG/GSH in glutathione peroxidase 4 (GPX4, an essential antioxidant enzyme) overexpressed aged transgenic mice.^8^ Similar results of down‐modulated oxidative stress mediators by DR or caloric restriction were also previously reported in normal aging human [S23, S24]. Simultaneously, decreased LA induced down‐modulation of its downstream ω6 PUFAs, that is, 9‐HODE, GLA, DGLA, and AA. In which 9‐HODE was converted through lipoxygenases (LOX) pathway [S25, S26], and also reported significantly downregulated in GPX4‐overexpressed aged mice compared with wild type animals.^8^ AA is the dominant fatty acid in skeletal muscle tissue constituting 10.5% in muscle lipids [S27] and 17.2% in muscle phospholipids [S28]. Downregulated AA and oxidative stress further contributed to the lower generation of other pro‐inflammatory mediators^11^ converted by cyclooxygenases (COX)^8^, ^10^ [S29, S30] (i.e., PGE_2_, PGD_2_, TXB_2_, and PGF_2α_), and LOX (i.e., 11‐HETE), which were all reported significantly (*P* < 0.05) higher in aged mice skeletal muscle compared with tissue of young mice^11^ and consistent with the notably (*P* < 0.05) down‐modulated PGE_2_, PGD_2_ and PGF_2α_ levels in GPX4‐overexpressed aged mice.^8^ The downregulated pro‐inflammatory mediators may contribute to diminished adverse effects for muscle cell/tissue function (Figure 6). PGF_2α_ and PGE_2_ are muscle growth and regeneration stimulator, respectively [S2–S4]. Downregulated PGF_2α_ and PGE_2_ may also contribute to decreased muscle mass (Figure 1B) by DR, and may support the theory of temporarily suppressing growth to redirect energy sources towards resilience, maintenance and (anti‐oxidant) defence mechanisms [17, S31, S32]. An association between TXB_2_ and ROS production in sarcopenic muscle is supported by.^11^ Downregulated TXB_2_ by DR may also contribute to lower ROS generation in sarcopenia muscle tissue. Reduced pro‐inflammation by caloric restriction is in line with literature of physiological aging, stressing the parallels between accelerated and natural aging and the anti‐aging effect of DR [S5, S33, S34].

Interestingly, even though there is a significant (*P* < 0.01) decrease of DGLA and AA, their downstream anti‐inflammatory products, that is, PGF_1_ and 8,9‐EET [S9], were notably increased by DR, which were converted through COX‐2 and cytochrome P450 (CYP) pathways [S25, S35, S36], respectively (Figure 6). ALA and its downstream ω3 PUFAs (i.e., EPA, DPA, and DHA) were also notably (*P* < 0.05) reduced by DR, however, some anti‐inflammatory mediators generated from EPA and ALA, and produced through CYP and soluble epoxide hydrolase (sEH) pathways [S26, S37], that is, 14,15‐DiHETE [S10] and 12,13‐DiHODE [S11], were significantly (*P* < 0.05) upregulated by DR. Enhanced anti‐inflammatory mediators by caloric restriction, including β‐hydroxybutyrate, were also reported by González et al. [S5] and Carlos et al.^16^ John et al. observed a strong correlation between increased β‐hydroxybutyrate concentration and reduced mortality (and improved memory) in aging mice with ketogenic diet [S38]. GSH, an antioxidant [S39], significantly (*P* < 0.01) increased, also indicating the improved anti‐inflammation by DR in *Ercc1* ^*∆/−*^ and *Xpg* ^*−/−*^ mice (Figure 4E), which was consistent with the increased GSH in an antioxidant enzyme overexpressed aged transgenic mouse model.^8^ The upregulated 8,9‐EET, 14,15‐DiHETE, and 12,13‐DiHODE, and their downregulated precursors indicate a possible modulation of CYP pathways by DR (Figure 6). The increased PGF_1_ and decreased PGE_2_, PGD_2_, PGF_2α_ and TXB_2_ may be due to the different types of COX involved, and a possible modulation of COX‐2 pathway by DR (Figure 6). CYP and COX‐2 could be potential biomarkers and pathways for attenuating sarcopenia. LOX pathway may not be affected by DR, or at least was not modulated to the level affecting its downstream metabolites, that is, 9‐HODE and 11‐HETE (Figure 6). Further proteomics and/or transcriptomics studies could be done on these pathways to further investigate DR effects in the future. The upregulated anti‐inflammatory and downregulated pro‐inflammatory mediators resulted in lower chronic inflammation in the progeroid mice, which in combination with the lower oxidative stress and lower energy expenditure contributed to improved muscle quality and performance (Figure 6) [S40–S42]. Similar results of reduced energy costs and ROS production by DR in humans were also reported by [S43–S45].

It is important to note that DNA‐repair‐deficient *Ercc1* ^*∆/−*^ mice are extremely sensitive to dietary PUFAs and live shorter when administered a high PUFA diet [S46]. This suggests that PUFAs elevate endogenous DNA damage, potentially via iron‐dependent lipid peroxidation [S46, S47] and aldehyde formation enhancing DNA‐damage‐induced transcription stress^27^ [S48, S49]. Reducing endogenous metabolites with DNA‐damaging capacities, like ω6 PUFAs, and potentially downstream aldehydes, could contribute to the mechanism of action by which DR lowers transcription stress.^17^ Enhanced energy production, lower energy expenditure, and reduced DNA damage and consequent transcription stress by DR likely all contribute to the dramatically extended healthspan and lifespan of progeroid mouse, including improved muscle quality, unaffected grip strength and improved motor coordination and learning performance by DR in *Ercc1* ^*∆/−*^ and *Xpg* ^*−/−*^ mice. Similar processes were recently also observed in healthy human volunteers following a 2‐year caloric restriction regimen.^20^ This comprehensive analysis provides fundamental metabolomics insights into the anti‐aging effects of DR on sarcopenia, and represents a snapshot in the progressive development of the premature aging phenotype at a relatively late stage. In the future it may be interesting to compare this to early stages to better understand the various intermediate steps leading to the development of sarcopenia along with human material. Further multi‐omics studies including transcriptomics (in relation with transcription stress) and proteomics (in combination with systemic circulating changes) to investigate the DR effects on muscle proteins and function may provide a more complete reference framework for future clinical therapy of sarcopenia.

## Conflict of interest

The authors declare no competing interests.

## Supporting information


**Data S1.** Supporting Information.

**Table S1.** The information of lipid ISTDs.
**Table S2.** The information of energy production related metabolites ISTDs.
**Table S3.** The information of analysed pro‐inflammatory, oxidative stress related, and muscle growth stimulation metabolites in mouse muscle samples.
**Table S4.** The information of analysed anti‐inflammatory metabolites in mouse muscle samples.
**Table S5.** The information of analysed energy production and storage related metabolites in mouse muscle samples.
