# Supplementary material for: Metabolomic analysis of dietary‐restriction‐induced attenuation of sarcopenia in prematurely aging DNA repair‐deficient mice
Source: J Cachexia Sarcopenia Muscle. 2024 Apr 30;15(3):868–82. doi: 10.1002/jcsm.13433 (PMC11154776; doi:10.1002/jcsm.13433)
Supplement: Supplementary file 2 — Table S1. The information of lipid ISTDs. Table S2. The information of energy production related metabolites ISTDs. Table S3. The information of analysed pro‐inflammatory, oxidative stress related, and muscle growth stimulation metabolites in mouse muscle samples. Table S4. The information of analysed anti‐inflammatory metabolites in mouse muscle samples. Table S5. The information of analysed energy production and storage related metabolites in mouse muscle samples. [file JCSM-15-868-s001.docx]

***Supplementary Information***

**Metabolomic analysis of dietary-restriction-induced attenuation of sarcopenia in prematurely aging DNA repair-deficient mice**

Yupeng He^1^, Wei Yang^1^, Luojiao Huang^1^, Marlien Admiraal-van Mever^1^, Rawi Ramautar^1^, Amy Harms^1^, Yvonne Rijksen^2,3^, Renata M.C. Brandt^4^, Sander Barnhoorn^4^, Kimberly Smit^2,3^, Dick Jaarsma^5^, Peter Lindenburg^1,6^, Jan H.J. Hoeijmakers^2,3,4,7^, Wilbert P. Vermeij^2,3^*, Thomas Hankemeier^1^*

^1^Metabolomics and Analytics Centre, Leiden Academic Centre for Drug Research, Leiden University, The Netherlands

^2^Princess Máxima Center for Pediatric Oncology, Utrecht, The Netherlands

^3^Oncode Institute, Utrecht, the Netherlands

^4^Department of Molecular Genetics, Erasmus MC Cancer Institute, Erasmus University Medical Center Rotterdam, Rotterdam, The Netherlands

^5^Department of Neuroscience, Erasmus University Medical Center Rotterdam, Rotterdam, The Netherlands

^6^Research Group Metabolomics, Leiden Center for Applied Bioscience, University of Applied Sciences Leiden, The Netherlands

^7^Institute for Genome Stability in Aging and Disease, Cologne Excellence Cluster for Cellular Stress Responses in Aging-Associated Diseases (CECAD), University of Cologne, Cologne, Germany

(*) corresponding authors

Wilbert P. Vermeij:

Heidelberglaan 25, 3584 CS Utrecht, The Netherlands

Tel.: +31 88 972 5266, E-mail: W.P.Vermeij@prinsesmaximacentrum.nl

Thomas Hankemeier:

Einsteinweg 55, 2333 CC Leiden, The Netherlands

Tel.: +31 71 527 1340, E-mail: hankemeier@lacdr.leidenuniv.nl

**Methods**

***Mouse models***

The generation and characterization of *Ercc1*^∆/+^ and *Ercc1*^+/−^ mice have been previously described [32]. *Ercc1*^∆/−^ mice were obtained by crossing *Ercc1*^∆/+^ (in a pure C57BL6J or FVB background) with *Ercc1*^+/−^ mice (in a pure FVB or C57BL6J background respectively) to yield *Ercc1*^∆/−^ offspring with a genetically uniform F1 C57BL6J/FVB hybrid background. *Xpg*^−/−^ mice have been generated and characterized previously [33] and were similarly obtained by crossing *Xpg*^+/−^ (in a pure C57BL6J background) with *Xpg*^+/−^ mice (in a pure FVB background). Wild-type (WT) F1 littermates (+/+ genotypes from *Ercc1*^∆/−^ and *Xpg*^−/−^ breedings) were used as controls. Hence, all animals used in the studies described here were of the same F1 C57BL6J/FVB hybrid genetic background. Typical unfavorable characteristics, such as blindness in an FVB background or deafness in a C57BL6J background, do not occur in this hybrid background. Only male mice were used in this paper to minimize the influence of gender on DR study.

All of the animals used were in accordance with the Principles of Laboratory Animal Care and with the guidelines approved by the Dutch Ethical Committee (permit no. 139-12-13, 139-12-18, 17-867-10, and 18-6886-05), in full accordance with European legislation.

***Behavioral analyses***

Motor coordination performance was assessed by measuring the average time spent on an (2-40 rpm) accelerating rotarod (Ugo Basile). All animals were given four consecutive trials of a maximum of 5 min with inter-trial intervals of 1 h. Grip strength was determined by placing mice with forelimbs or all limbs on a grid attached to a force gauge, and steadily pulling the mice by their tail. Grip strength is defined as the maximum strength produced by the mouse before releasing the grid. For each value the test was performed in triplicate. Behavioral assays (motor coordination and grip strength) were measured in the week at 16 weeks and 14 weeks of age for *Ercc1*^∆/−^ and *Xpg*^−/−^, respectively. 50% of WT mice were assessed and collected at the same time as the *Xpg*^−/−^ mice and 50% with the *Ercc1*^∆/−^ mice, but this two weeks difference did not yield into any trend difference in motor coordination performance nor grip strength. Body weight was also determined just before mice collection.

***Metabolomic analysis preparation***

*Chemicals and internal standards (ISTDs)*

Butylated hydroxytoluene (BHT) and methyl tert-butyl ether (MTBE) were obtained from Sigma-Aldrich (Steinheim, Germany). 1-butanol was purchased from Acros Organics (Geel, Belgium). MilliQ water was obtained from a Millipore high-purity water dispenser (Billerica, MA, USA). All solvents were HPLC grade or higher. For internal standards (ISTDs), stable isotope (deuterium-, carbon-, and/or nitrogen-) labelled metabolites were used. Labelled oxylipins, fatty acids, and endocannabinoids ISTDs were acquired from Cayman Chemicals (Ann Arbor, MI, USA) (Table S1). Labelled ATP, AMP and UTP were purchased from Sigma-Aldrich (Steinheim, Germany). Labelled amino acid and organic acids ISTDs were ordered from Cambridge Isotope Laboratories (Andover, MA, USA) (Table S2).

For the lipid ISTDs, the stock solution was prepared in MeOH in a stated concentration (Table S1) containing 0.4 mg/mL BHT . For the stock solution of metabolites related to energy production, the ISTDs were prepared in MilliQ water in stated concentration in Table S2.

*Muscle tissue extraction*

Muscle tissues were lyophilized in a CHRIST (Alpha 3-4 LSC basic) freeze-dryer (Osterode am Harz, Germany; connected to a Vacuubrand Chemistry Hybrid Pump RC6 high vacuum pump, Wertheim, Germany) for 24 hours, and weighed. 100 mg (± 10%) zirconium oxide beads (0.5 mm; Next Advance, Averill Park, NY, USA) were utilized for dry-homogenization of muscle tissues in a Bullet Blender (BBX24; Next Advance, Averill Park, NY, USA) for 15 min at speed 9. 10 µL labelled ISTDs of lipids (Table S1) and energy-production-related metabolites (Table S2) were spiked in the muscle tissues before extraction to correct for potential bias during sample extraction.

5 µL antioxidant solution (0.4mg/mL BHT:EDTA=1:1), 400 µL of cold MilliQ water, and 1 mL organic solvent (BuOH: MTBE=1:1, v/v) were added to all samples, and settled on ice for 20 min before the homogenization by Bullet Blender for 15 min at speed 9. Then the homogenized samples were centrifuged (2,000×g, 4 ℃) for 10 min. 900 µL and 200 µL of the upper organic and lower aqueous phase were collected, respectively, evaporated and reconstituted to 50 µL MeOH for organic phase and 100 µL 50% MeOH (50% MilliQ Water) for aqueous phase.

***Metabolite analysis***

*Lipid metabolite analysis*

Each sample was measured with two complementary reverse phase methods using mobile phases with different pH.

The low pH run utilized an Acquity BEH C18 column (50 × 2.1 mm, 1.7 μm; Waters, USA) on a Shimadzu LC-30AD (Japan) hyphenated to a SCIEX Q-Trap 6500+ (Framingham, MA, USA). Separations were performed using three mobile phases: (A) water with 0.1% acetic acid; (B) ACN: MeOH (9:1, v/v) with 0.1% acetic acid; (C) Isopropanol with 0.1% acetic acid at 40 °C at a flow rate of 0.7 mL/min. The 16 minute run used the following gradient: start with 20% B and 1% C; B was increased to 85% between 0.75 and 14 min, and C was increased to 15% between 11 and 14 min; the condition held for 0.5min prior to column re-equilibration at the starting conditions from 14.8 to 16 min. Data was acquired using Sciex Analyst software (Version 1.7, Framingham, MA, USA) and peak integration used Sciex OS (Version 1.4.0, Framingham, MA, USA).

The high pH run used a Kinetex® Core-Shell EVO 100 Å C18 column (50 × 2.1 mm, 1.8 μm; Phemomenex, USA) on a Shimadzu LCMS-8060 system (Shimadzu, Japan). Separations used mobile phases (A) 5% ACN with 2 mM ammonium acetate and 0.1% ammonium hydroxide and (B) 95% ACN with 2 mM ammonium acetate and 0.1% ammonium hydroxide at 40 °C at a flow rate of 0.6 mL/min. The gradient started with 1% B; B was increased to 100% from 0.7 to 7.7 min; 100% B held for 0.75 min prior to re-equilibration at the starting conditions between 8.75 and 11 min. Data was acquired and peaks integrated using LabSolutions (Version 5.97 SP1, Shimadzu, Japan). Multiple reaction monitoring (MRM) was utilized in MS/MS acquisition in both positive and negative electrospray ionization mode with polarity switching for the low and high pH method.

*Analysis of energy-production-related metabolites*

The energy-production-related metabolites were analyzed by a hydrophilic interaction liquid chromatography (HILIC) mass spectrometry platform. Briefly, a Waters UPLC (AcquityTM, Milford, MA, USA) coupled with a SeQuant ZIC- cHILIC column (PEEK 100 × 2.1 mm, 3.0 μm particle size; Merck KGaA, Darmstadt, Germany) at 30 °C and a Sciex MS (Triple-TOF 5600+, Framingham, MA, USA) were applied for separation using method: (A) 90% acetonitrile with 5 mM ammonium acetate at pH 6.8; (B) 10% acetonitrile with 5 mM ammonium acetate at pH 6.8, at a flow rate of 0.25 mL/min with the gradient method: 100% A for 2 min; ramping 3–20 min to 60% A; ramping 20–20.1 min to 100% A and re-equilibrated to 35 min with 100% A. The MS data was acquired at full scan range 50–900 m/z in negative ionization mode with curtain gas 39.3 psi, source temperature 400 °C and ion source voltage 4.64 kV by Sciex Analyst (Version 1.7, Framingham, MA, USA) and peak integration used Sciex OS (Version 1.4.0, Framingham, MA, USA). β-hydroxybutyrate, reduced glutathione (GSH) and oxidized glutathione (GSSG) were also analyzed by HILIC-MS method.

**Table S1.** The information of lipid ISTDs

| **Name** | **Concentration(mM)** | **Precursor Mass (M/Z)** | **Fragment Mass (M/Z)** | **Retention Time (min)** |
| --- | --- | --- | --- | --- |
| 10-NO_2_-OA-d_17_ | 2.90 | 343.2 | 183.2 | 13.2 |
| C18:1-ω9-d_17_ | 3.34 | 298.1 | 298.1 | 13.8 |
| C18:2-ω6-d_4_ | 3.34 | 283.2 | 265.201 | 13.6 |
| C22:6-ω3-d_5_ | 1.50 | 332.1 | 288.4 | 13.4 |
| C20:4-ω6-d_8_ | 32.00 | 311.1 | 267.2 | 13.47 |
| 14,15-DiHETrE-d_11_ | 0.29 | 348.2 | 207.1 | 9.8 |
| 5-iPF_2α_-VI-d_11_ | 0.27 | 364.2 | 115.05 | 3.9 |
| 8,12-iPF_2α_-IV-d_11_ | 0.27 | 364.21 | 115.05 | 5.9 |
| 12,13-DiHOME-d_4_ | 0.31 | 317.2 | 185.1 | 9.3 |
| 8iso-PGE_2_-d_4_ | 0.28 | 355.3 | 275.25 | 5.42 |
| 8iso-PGF_2α_-d_4_ | 0.28 | 357.3 | 197.15 | 4.75 |
| 9,10-DiHOME-d_4_ | 0.31 | 317.2 | 203.1 | 9.5 |
| 9-HODE-d_4_ | 0.33 | 299.2 | 172.1 | 11.1 |
| LTB_4_-d_4_ | 0.29 | 339.5 | 197.1 | 9.2 |
| PGE_2_-d_4_ | 0.28 | 355.3 | 275.25 | 4.8 |
| PGF_2α_-d_4_ | 0.28 | 357.3 | 197.15 | 4.75 |
| TXB_2_-d_4_ | 0.27 | 373.5 | 173.3 | 3.7 |
| 20-HETE-d_6_ | 0.31 | 325.2 | 279.2 | 10.6 |
| 12-HETE-d_8_ | 0.30 | 327.2 | 184.1 | 11.7 |
| 5-HETE-d_8_ | 0.30 | 327.1 | 116.15 | 12.1 |

**Table S2.** The information of energy production related metabolites ISTDs

| **Name** | **Concentration** | **Molar mass (M/Z)** | **Retention Time (min)** |
| --- | --- | --- | --- |
| ATP-^13^C_10_, ^15^N_5_ | 10 mg/mL | 520.9885 | 8.0 |
| AMP-^13^C_10_, ^15^N_5_ | 10 mg/mL | 361.0558 | 4.2 |
| Pyruvate-^13^C_3_ | 500 mM | 90.0188 | 2.2 |
| Succinate-d_4_ | 500 mM | 121.0444 | 3.7 |
| UMP-^15^N_2_ | 500 mM | 325.0227 | 4.4 |
| Valine-^13^C_5_ | 0.5 mg/mL | 123.0863 | 3.5 |

**Table S3.** The information of analyzed pro-inflammatory, oxidative stress related, and muscle growth stimulation metabolites in mouse muscle samples

| **Name** | **Precursor Mass (M/Z)** | **Fragment Mass (M/Z)** | **Retention Time (min)** | **ISTD** | **ChEBI ID** |
| --- | --- | --- | --- | --- | --- |
| ***Analyzed by LC-MS/MS method for lipid metabolites (in section 2.6.1)*** | | | | | |
| LA (C18:2-ω6) | 279.2 | 261.2 | 13.6 | C18:2-ω6-d_4_ | 17351 |
| GLA (C18:3-ω6) | 277 | 233.001 | 13.31 | C18:2-ω6-d_4_ | 28661 |
| DGLA (C20:3-ω6) | 305.1 | 261 | 13.7 | C20:4-ω6-d_8_ | NA |
| AA (C20:4-ω6) | 303 | 259 | 13.5 | C20:4-ω6-d_8_ | 15843 |
| AdA (C22:4-ω6) | 331.5 | 287.5 | 13.8 | C22:6-ω3-d_5_ | NA |
| PGD_2_ | 351.1 | 271.15 | 5.1 | PGE_2_-d_4_ | 15555 |
| PGE_2_ | 351.1 | 271.15 | 4.8 | PGE_2_-d_4_ | 15551 |
| PGF_2α_ | 353.1 | 193.1 | 4.6 | PGF_2α_ -d_4_ | 15553 |
| TXB_2_ | 369.2 | 169.1 | 3.8 | TXB_2_-d_4_ | 28728 |
| 9-HODE | 295.21 | 171.1 | 11.1 | 9-HODE-d_4_ | 72651 |
| 13-HODE | 295.2 | 195.2 | 11 | 9-HODE-d_4_ | 72639 |
| 12(S)-HETrE | 279.2 | 179.1 | 9.8 | 12-HETE-d_8_ | 90771 |
| 11-HETE | 319.2 | 167.1 | 11.6 | 12-HETE-d_8_ | 72606 |
| 15-HETE | 319.2 | 219.2 | 11.3 | 5-HETE-d_8_ | 64017 |
| 20-HETE | 319.2 | 289.2 | 10.6 | 20-HETE-d_6_ | 34306 |
| 5-HETE | 319.2 | 115.15 | 12.3 | 5-HETE-d_8_ | 28209 |
| 8-HETE | 319.2 | 155.1 | 11.8 | 5-HETE-d_8_ | 34486 |
| 9-HETE | 319.21 | 167.1 | 12 | 12-HETE-d_8_ | 72786 |
| 12-HETE | 319.2 | 179.2 | 11.8 | 12-HETE-d_8_ | 19138 |
| ***Analyzed by HILIC-MS method (in section 2.6.2)*** | | | | | |
| GSH | 306.0765 (M/Z) | | 4.5 | Succinate-d_4_ | 16856 |
| GSSG | 611.1447 (M/Z) | | 7.4 | Succinate-d_4_ | 17858 |

**Table S4.** The information of analyzed anti-inflammatory metabolites in mouse muscle samples.

| **Name** | **Precursor Mass (M/Z)** | **Fragment Mass (M/Z)** | **Retention Time (min)** | **ISTD** | **ChEBI ID** |
| --- | --- | --- | --- | --- | --- |
| ***Analyzed by LC-MS/MS method for lipid metabolites (in section 2.6.1)*** | | | | | |
| ALA (C18:3-ω3) | 277.1 | 233.15 | 3.9 | C18:2-ω6-d_4_ | 27432 |
| EPA (C20:5-ω3) | 301.1 | 257.2 | 13.3 | C20:4-ω6-d_8_ | 28364 |
| DPA (C22:5-ω3) | 329.2 | 285.4 | 4.7 | C22:6-ω3-d_5_ | NA |
| DHA (C22:6-ω3) | 327.1 | 283.1 | 13.4 | C22:6-ω3-d_5_ | 28125 |
| 12,13-DiHODE | 311.2 | 293.15 | 8.46 | 9-HODE-d_4_ | 88461 |
| 14,15-DiHETE | 335.2 | 207.1 | 9.1 | 14,15-DiHETrE-d_11_ | 88459 |
| 17,18-DiHETE | 335.2 | 247.1 | 8.9 | 14,15-DiHETrE-d_11_ | 88349 |
| TXB_1_ | 371.2 | 171.1 | 3.3 | TXB_2_-d_4_ | 73994 |
| PGF_1_ | 355.2 | 311.1 | 4.75 | PGF_2α_-d_4_ | 28852 |
| 11,12-EET | 319.22 | 167.1 | 12.8 | 14,15-DiHETrE-d_11_ | 34130 |
| 14,15-EET | 319.21 | 219.2 | 12.5 | 14,15-DiHETrE-d_11_ | 34157 |
| 8,9-EET | 319.21 | 155.1 | 12.8 | 14,15-DiHETrE-d_11_ | 34490 |
| 12-HEPE | 317.2 | 179.1 | 10.8 | 12-HETE-d8 | 88345 |
| 15-HEPE | 317.2 | 219.2 | 10.6 | 5-HETE-d_8_ | 72627 |
| 18-HEPE | 317.2 | 299.2 | 10.4 | 12-HETE-d8 | 72802 |
| 5-HEPE | 317.2 | 115.2 | 11.1 | 5-HETE-d_8_ | 72801 |
| 9-HEPE | 317.2 | 167.25 | 10.9 | 12-HETE-d_8_ | 89570 |
| PGE_1_ | 353.2 | 317.2 | 5.15 | PGE_2_-d_4_ | 15544 |
| PGE_3_ | 349.2 | 269.2 | 3.34 | PGE_2_-d_4_ | 28031 |
| ***Analyzed by HILIC-MS method (in section 2.6.2)*** | | | | | |
| β-hydroxybutyrate | 103.0401 (M/Z) | | 2.3 | Pyruvate-^13^C_3_ | 20067 |

**Table S5.** The information of analyzed energy production and storage related metabolites in mouse muscle samples

| **Metabolites name** | **ChEBI ID** | **Detected Mass (M/Z)** | **Retention time (min)** | **ISTD** | | |
| --- | --- | --- | --- | --- | --- | --- |
| ***Analyzed by LC-MS/MS method for lipid metabolites (in section 2.6.1)*** | | | | | |  |
| Myristic acid (C14:0) | 28875 | 227.2>209.2 | 11.6 | C18:1-ω9-d_17_ | | |
| Palmitic acid (C16:0) | 15756 | 255.2>237.2 | 13.8 | C18:1-ω9-d_17_ | | |
| Stearic acid (C18:0) | 28842 | 283.2>265.2 | 14.1 | C18:1-ω9-d_17_ | | |
| ***Analyzed by HILIC-MS method (in section 2.6.2)*** | | | | |  |  |
| Glucose | 17234 | 179.0561 | 4.0 | Succinate-d_4_ | | |
| Glucose-1-P | 58601 | 259.0224 | 5.9 | Succinate-d_4_ | | |
| Glucose-6-P | 14314 | 259.0224 | 6.5 | Succinate-d_4_ | | |
| Fructose-6-P | 78697 | 259.0224 | 6.2 | Succinate-d_4_ | | |
| Pyruvate | 15361 | 87.0088 | 2.2 | Pyruvate-^13^C_3_ | | |
| Acetyl-CoA | 15351 | 808.1185 | 0.9 | Succinate-d_4_ | | |
| Citrate | 16947 | 191.0919 | 8.1 | Succinate-d_4_ | | |
| Cis-Aconitate | 16383 | 173.0085 | 3.8 | Pyruvate-^13^C_3_ | | |
| α-Ketoglutarate | 80619 | 145.0142 | 3.9 | Succinate-d_4_ | | |
| Succinate | 30779 | 117.0193 | 3.7 | Succinate-d_4_ | | |
| Succinyl-CoA | 15380 | 866.1312 | 4.8 | Succinate-d_4_ | | |
| Malate | 25115 | 133.0142 | 5.1 | Succinate-d_4_ | | |
| Fumarate | 37154 | 115.0037 | 4.4 | Succinate-d_4_ | | |
| Alanine | 15570 | 90.0550 | 4.6 | Valine-^13^C_5_ | | |
| Phosphocreatine | 17287 | 210.0285 | 6.1 | Succinate-d_4_ | | |
| Creatine | 16919 | 130.0622 | 4.6 | Succinate-d_4_ | | |
| ATP | 15422 | 505.9885 | 8.0 | ATP-^13^C_10_, ^15^N_5_ | | |
| ADP | 16761 | 426.0221 | 7.2 | AMP-^13^C_10_, ^15^N_5_ | | |
| AMP | 16027 | 346.0558 | 4.2 | AMP-^13^C_10_, ^15^N_5_ | | |
| 6-phosphogluconate | 48928 | 275.0174 | 7.1 | Succinate-d_4_ | | |
| Riboluse-5-P | 17363 | 229.0118 | 4.9 | Succinate-d_4_ | | |
| Ribose-5-P | 78679 | 229.0119 | 5.1 | Succinate-d_4_ | | |

**References**

1. Wang L, Chen P, Xiao W. β-hydroxybutyrate as an Anti-Aging Metabolite. Nutrients. 2021;**13**:3420.
2. Horsley V, Pavlath GK. Prostaglandin F2(alpha) stimulates growth of skeletal muscle cells via an NFATC2-dependent pathway. J Cell Biol. 2003;**161**:111-8.
3. Jansen KM, Pavlath GK. Prostaglandin F2α promotes muscle cell survival and growth through upregulation of the inhibitor of apoptosis protein BRUCE. Cell Death & Differentiation. 2008;**15**:1619-28.
4. Ho ATV, Palla AR, Blake MR, Yucel ND, Wang YX, Magnusson KEG, et al. Prostaglandin E2 is essential for efficacious skeletal muscle stem-cell function, augmenting regeneration and strength. Proceedings of the National Academy of Sciences of the United States of America. 2017;**114**:6675-84.
5. González OA, Tobia C, Ebersole JL, Novak MJ. Caloric restriction and chronic inflammatory diseases. Oral diseases. 2012;**18**:16-31.
6. Milanese C, Bombardieri CR, Sepe S, Barnhoorn S, Payán-Goméz C, Caruso D, et al. DNA damage and transcription stress cause ATP-mediated redesign of metabolism and potentiation of anti-oxidant buffering. Nat Commun. 2019;**10**:4887.
7. Calder PC. n-3 fatty acids and cardiovascular disease: evidence explained and mechanisms explored. Clinical Science. 2004;**107**:1-11.
8. Fan YY, Chapkin RS. Importance of dietary gamma-linolenic acid in human health and nutrition. Journal of Nutrition. 1998;**128**:1411-4.
9. Morisseau C, Hammock BD. Impact of soluble epoxide hydrolase and epoxyeicosanoids on human health. Annual review of pharmacology and toxicology. 2013;**53**:37-58.
10. Cioccari L, Luethi N, Duong T, Ryan E, Cutuli SL, Lloyd-Donald P, et al. Cytokine and lipid metabolome effects of low-dose acetylsalicylic acid in critically ill patients with systemic inflammation: a pilot, feasibility, multicentre, randomised, placebo-controlled trial. Critical Care and Resuscitation. 2020;**22**:227-36.
11. West AL, von Gerichten J, Irvine NA, Miles EA, Lillycrop KA, Calder PC, et al. Fatty acid composition and metabolic partitioning of α-linolenic acid are contingent on life stage in human CD3(+) T lymphocytes. Front Immunol. 2022;**13**:1079642.
12. Norman K, Stobäus N, Lochs H, Pirlich M. Measurement of hand grip strength as nutritional outcome parameter. Aktuelle Ernährungsmedizin. 2009;**34**:263-8.
13. Rhoades RA, Bell DR. Medical phisiology: Principles for clinical medicine. Lippincott Williams & Wilkins; 2012.
14. DeFronzo RA, Tripathy D. Skeletal muscle insulin resistance is the primary defect in type 2 diabetes. Diabetes care. 2009;**32 Suppl 2**:S157-S63.
15. Xie W-Q, Xiao W-F, Tang K, Wu Y-X, Hu P-W, Li Y-S, et al. Caloric restriction: implications for sarcopenia and potential mechanisms. Aging (Albany NY). 2020;**12**:24441-52.
16. Salway JG. Metabolism at a Glance. John Wiley & Sons; 2016.
17. Ingram DK, Weindruch R, Spangler EL, Freeman JR, Walford RL. Dietary Restriction Benefits Learning and Motor Performance of Aged Mice. Journal of Gerontology. 1987;**42**:78-81.
18. Asadi Shahmirzadi A, Edgar D, Liao CY, Hsu YM, Lucanic M, Asadi Shahmirzadi A, et al. Alpha-Ketoglutarate, an Endogenous Metabolite, Extends Lifespan and Compresses Morbidity in Aging Mice. Cell metabolism. 2020;**32**:447-56.
19. Spiteller G. Is Lipid Peroxidation of Polyunsaturated Acids the Only Source of Free Radicals That Induce Aging and Age-Related Diseases? Rejuvenation Research. 2010;**13**:91-103.
20. Macsai M, Mojica G. 34 - Medical Management of Ocular Surface Disease. In: Holland EJ, Mannis MJ, Lee WB editors. London: W.B. Saunders; 2013;271-81.
21. Kitson AP, Smith TL, Marks KA, Stark KD. Tissue-specific sex differences in docosahexaenoic acid and Delta6-desaturase in rats fed a standard chow diet. Appl Physiol Nutr Metab. 2012;**37**:1200-11.
22. Hofer T, Marzetti E, Xu J, Seo AY, Gulec S, Knutson MD, et al. Increased iron content and RNA oxidative damage in skeletal muscle with aging and disuse atrophy. Experimental gerontology. 2008;**43**:563-70.
23. Redman LM, Smith SR, Burton JH, Martin CK, Il'yasova D, Ravussin E. Metabolic Slowing and Reduced Oxidative Damage with Sustained Caloric Restriction Support the Rate of Living and Oxidative Damage Theories of Aging. Cell metabolism. 2018;**27**: 805–815.
24. Sutton EF, Beyl R, Early KS, Cefalu WT, Ravussin E, Peterson CM. Early Time-Restricted Feeding Improves Insulin Sensitivity, Blood Pressure, and Oxidative Stress Even without Weight Loss in Men with Prediabetes. Cell metabolism. 2018;**27**:1212-21.
25. Svenvik M, Raffetseder J, Brudin L, Lindberg R, Blomberg M, Axelsson D, et al. Plasma oxylipin levels associated with preterm birth in preterm labor✰. Prostaglandins, Leukotrienes and Essential Fatty Acids. 2021;**166**:102251.
26. Rajamani A, Borkowski K, Akre S, Fernandez A, Newman JW, Simon SI, et al. Oxylipins in triglyceride-rich lipoproteins of dyslipidemic subjects promote endothelial inflammation following a high fat meal. Sci Rep. 2019;**9**:8655.
27. McGlory C, Galloway SD, Hamilton DL, McClintock C, Breen L, Dick JR, et al. Temporal changes in human skeletal muscle and blood lipid composition with fish oil supplementation. Prostaglandins Leukot Essent Fatty Acids. 2014;**90**:199-206.
28. Smith GI, Atherton P, Reeds DN, Mohammed BS, Rankin D, Rennie MJ, et al. Omega-3 polyunsaturated fatty acids augment the muscle protein anabolic response to hyperinsulinaemia-hyperaminoacidaemia in healthy young and middle-aged men and women. Clin Sci (Lond). 2011;**121**:267-78.
29. Nørregaard R, Kwon TH, Frøkiær J. Physiology and pathophysiology of cyclooxygenase-2 and prostaglandin E2 in the kidney. Kidney Res Clin Pract. 2015;**34**:194-200.
30. Das UN. Ageing: Is there a role for arachidonic acid and other bioactive lipids? A review. Journal of Advanced Research. 2018;**11**:67-79.
31. Schumacher B, van der Pluijm I, Moorhouse MJ, Kosteas T, Robinson AR, Suh Y, et al. Delayed and accelerated aging share common longevity assurance mechanisms. PLoS genetics. 2008;**4**:e1000161.
32. Finkel T. The metabolic regulation of aging. Nature medicine. 2015;**21**:1416-23.
33. Ma S, Sun S, Geng L, Song M, Wang W, Ye Y, et al. Caloric Restriction Reprograms the Single-Cell Transcriptional Landscape of Rattus Norvegicus Aging. Cell. 2020;**180**:984-1001.
34. Lijnen HR, Van Hul M, Hemmeryckx B. Caloric restriction improves coagulation and inflammation profile in obese mice. Thrombosis research. 2012;**129**:74-9.
35. Xiao Y, Gu Y, Purwaha P, Ni K, Law B, Mallik S, et al. Characterization of free radicals formed from COX-catalyzed DGLA peroxidation. Free radical biology & medicine. 2011;**50**:1163-70.
36. Wang B, Wu L, Chen J, Dong L, Chen C, Wen Z, et al. Metabolism pathways of arachidonic acids: mechanisms and potential therapeutic targets. Signal Transduct Target Ther. 2021;**6**:94.
37. Borsini A, Nicolaou A, Camacho-Muñoz D, Kendall AC, Di Benedetto MG, Giacobbe J, et al. Omega-3 polyunsaturated fatty acids protect against inflammation through production of LOX and CYP450 lipid mediators: relevance for major depression and for human hippocampal neurogenesis. Mol Psychiatry. 2021;**26**:6773-88.
38. Newman JC, Covarrubias AJ, Zhao M, Yu X, Gut P, Ng C-P, et al. Ketogenic Diet Reduces Midlife Mortality and Improves Memory in Aging Mice. Cell metabolism. 2017;**26**:547-57.
39. Aquilano K, Baldelli S, Ciriolo MR. Glutathione: new roles in redox signaling for an old antioxidant. Frontiers in pharmacology. 2014;**5**:196.
40. Wang H, Ye J. Regulation of energy balance by inflammation: common theme in physiology and pathology. Rev Endocr Metab Disord. 2015;**16**:47-54.
41. Floh AA, Nakada M, La Rotta G, Mah K, Herridge JE, Van Arsdell G, et al. Systemic Inflammation Increases Energy Expenditure Following Pediatric Cardiopulmonary Bypass. Pediatric Critical Care Medicine. 2015;**16**:343-51.
42. He M, Harms AC, van Wijk E, Wang M, Berger R, Koval S, et al. Role of amino acids in rheumatoid arthritis studied by metabolomics. Int J Rheum Dis. 2019;**22**:38-46.
43. Heilbronn LK, de Jonge L, Frisard MI, DeLany JP, Larson-Meyer DE, Rood J, et al. Effect of 6-month calorie restriction on biomarkers of longevity, metabolic adaptation, and oxidative stress in overweight individuals: a randomized controlled trial. JAMA. 2006;**295**:1539-48.
44. Almundarij TI, Gavini CK, Novak CM. Suppressed sympathetic outflow to skeletal muscle, muscle thermogenesis, and activity energy expenditure with calorie restriction. Physiol Rep. 2017;**5**:e13171.
45. Civitarese AE, Carling S, Heilbronn LK, Hulver MH, Ukropcova B, Deutsch WA, et al. Calorie restriction increases muscle mitochondrial biogenesis in healthy humans. PLoS Med. 2007;**4**:e76.
46. Czerwińska J, Nowak M, Wojtczak P, Dziuban-Lech D, Cieśla JM, Kołata D, et al. ERCC1-deficient cells and mice are hypersensitive to lipid peroxidation. Free radical biology & medicine. 2018;**124**:79-96.
47. Jenkinson AM, Collins AR, Duthie SJ, Wahle KW, Duthie GG. The effect of increased intakes of polyunsaturated fatty acids and vitamin E on DNA damage in human lymphocytes. FASEB journal : official publication of the Federation of American Societies for Experimental Biology. 1999;**13**:2138-42.
48. Mulderrig L, Garaycoechea JI, Tuong ZK, Millington CL, Dingler FA, Ferdinand JR, et al. Aldehyde-driven transcriptional stress triggers an anorexic DNA damage response. Nature. 2021;**600**:158-63.
49. Wang M, Dingler FA, Patel KJ. Genotoxic aldehydes in the hematopoietic system. Blood. 2022;**139**:2119-2129.
50. von Haehling S, Coats AJ, Anker SD. Ethical guidelines for publishing in the Journal of Cachexia, Sarcopenia and Muscle: update 2021. Wiley Online Library; 2021;2259-61.
